# Some Remarks on Fracture of the Head of the Femur

**Published:** 1905-08

**Authors:** T. J. Bennett

**Affiliations:** Austin, Texas


TEXAS MEDICAL JOURNAL.

ESTABLISHED JULY, 1885.

PUBLISHED MONTHLY.—SUBSCRIPTION $1.00 A YEAR.

Vol. XXI. AUSTIN, AUGUST, 1905. No. 2.

For Texas Medical Journal.

### Some Remarks on Fracture of the Head of the Femur.

BY T. J. BENNETT, M. D., AUSTIN, TEXAS.

Eminent authorities regard this fracture as one of the most difficult to successfully treat of any in the entire list. It is always and justly looked upon as being an injury that is not only serious to the patient, but also serious to the reputation of the surgeon in charge. There is not a community in perhaps any country that has not a few permanent cripples as monuments to the profession's discredit. I am ashamed to acknowledge that mistakes in the grosser diagnosis of injuries at the hip joint are not so very uncommon, and I point with an unusual degree of embarrassment to several such meandering about in my own bailiwick. But Senn and other good authorities say that there are cases where no one is to blame for the bad results, but it is not always certain even in these cases that the attending surgeon's reputation does not suffer. Such results are too often viewed in the light of the strictly business laity: "His hip was broken; he was treated by So and So; he is now a criple for life." The reason of the failure is difficult to explain because it may be due to several causes instead of the one dependent upon the skill of the physician, and may not be known to anyone until time alone has given the result. But in time, as the victim hobbles through life, the particular surgeon may be lost sight of, but the profession at large will carry the odium through the succeeding generation.

It is not necessary to enumerate the individual causes of fracture of the head of the femur. Let it suffice, therefore, for as brief a discussion as this, to state that sufficient force applied (1) through the axis of the femur, or (2) horizontally over the great trochanter in the axis of the femoral neck, or (3) by certain traction force accompanied by extreme adduction of the limb, or (4) extreme rotation would produce this fracture. It has been shown that the most frequent cause of the fracture is the application of force in the direction of the neck of the femur by side-falls upon the trochanter, and that the exact part most frequently broken is the trochanteric portion partially within and partially without the capsule.

The question of exact diagnosis, however, is not always the easiest thing in the world to settle—in fact, it is upon this point that failure in the result is so often dependent. The grosser diagnosis as to fracture or no fracture, and whether the injury is an impacted fracture, should always be made out, and the diagnosis as to intra or extra capsular fracture or fracture involving the capsule itself, should be approximated as nearly as possible. Statistics show great unreliability as to the exact nature of these fractures. The X-rays have done a great deal in clearing the hazy points in the diagnosis. I believe the grosser diagnosis of fracture, or no fracture, should be always made, and if necessary a general anesthetic should be administered to determine the point.

The differentiation between an impacted fracture and a non-impacted fracture, it matters not whether the latter is intra or extra capsular or mixed, is the first and most important question to settle, because in the former manipulation should be made with care lest the impaction should be disturbed and a failure in union be rendered probable. The treatment in both instances is practically the same, except that Buck's extention is not employed in cases of impaction, whereas it may or may not be used in the simple fracture, depending upon the kind of splint made use of in the treatment. The impacted fracture demands to be put as found. It can usually be determined to be an impacted fracture by finding decided shortening of the limb, eversion of the foot or other deformity, and by there being more or less marked immobility at the head of the femur. The simple fracture in contrast can nearly always be determined by the presence of deformity, lost motion and crepitation. The deformity as relates to length of limb and other malposition should be manipulated into as nearly the normal position as possible, and there fixed by a suitable splint.

The best authorities, where any opinion is expressed at all, agree that a fracture may be expected in practically all cases where the patient is beyond 60 years of age and has sustained a side-fall with resulting loss of function of the limb. This view accords with my limited experience in this class of cases.

It is necessary also to differentiate between fracture of the head of the femur and other injuries, such as dislocation, severe sprain and bruises of the muscles and ligaments in this region.

It is claimed that the capsular ligament is always stronger than the bone after 55 or 60 years of age, hence a fracture is more liable to occur than a dislocation at that time of life. And per contra, the bone being stronger earlier in life than the capsular ligament, dislocation is more frequently observed at that age than fracture. The same may be said of sprains and severe bruises. The old person receiving a blow sufficiently great to cause sprain or bruise is liable to produce fracture, whereas the young person receiving such injury is more liable to sprain or bruise.

If you will pardon me for being personal, I will say that I am a victim of fracture of the head femoral neck myself, and as far as a personal experience goes, I am in position to state that the treatment is indeed a most serious ordeal. The fracture in my case was caused by a side-fall against the ground from overturning my automobile while attempting to avoid a collision with a messenger boy on a bicycle. We were coming meeting each other, both very near the center of the street. I turned to the right and instead of his turning to his right he turned to his left. Upon seeing that he was not going to miss me I wheeled to my left, and instantly the boy did the same thing, that is, he turned to his right, which, in order to avoid the chance of killing him, I was crowded off the street onto a slight grade, which had the effect to overturn the vehicle. The boy missed the automobile entirely, and, strange to say, the little rascal has never seen fit to reveal his identity to this day. There were two young men in the auto with me at the time of the accident, one escaped injury entirely and the other sustained a Colles fracture.

I was removed to my residence, where a general anesthetic was administered and the diagnosis of fracture of the femoral neck was made. A long side-splint was applied, reaching from the foot of the axilla. Extension was made with a fifteen-pound weight, and sand bags, five or six inches in diameter, were placed along side the limb, inside as well as outside, and one against the opposite trochanter. In this position I lay, fully appreciating the gravity of the case, for eight weeks, not deviating as much as an inch to the right or to the left. Here is where the ordeal came in. A special Thomas splint was tried, but it was too uncomfortable and had to be abandoned. It was about the same as sleeping on a fence rail. Plaster of Paris was practically out of the question in my case on account of my weight, which at the time was something over 220 pounds. The result, I am glad to say, has been good, but it required more time than is generally supposed in such cases. The weight of the body being sustained at almost a right angle to the attachment of the shaft above the attempt to walk causes a real risk for the longest time, and especially does it create a monstrous fear that something is going to give way if one does walk. It has been six months since the accident, and I am just beginning to use my leg in walking. There is no shortening and I have perfect union. This is a better result than I have ever obtained in any case of this injury. But maybe I was a better patient than the average.

The principle in the treatment of this fracture is identical with that of treating any other fracture, and consists in properly adjusting the fractured ends of the bone and securely holding them in the natural corrected position. There is not anything in the bone itself which differs from a fractured bone anywhere else in the body. The chief trouble is encountered in properly holding the fragments of broken bone in position. The intracapsular fracture presents the greatest difficulty and is quite often, despite the most skillful efforts, a failure in the result. The callus in this fracture is confined to the inner surface between the ends of the bone, the capsule preventing the formation of callus on the otuside in a manner to give support.

The treatment which seems to have the greatest number of authoritative advocates is the plaster of Paris splint. This is applied so as to include the injured limb from the toes to the ensiform cartilage together with the pelvis and the well limb down to the knee. Senn's plan is to apply the plaster on the injured limb from the toes to half way the thigh, and then have the patient held up in the erect position by two strong men, the patient standing on his well foot on a box a suitable height from the floor. The injured limb is placed in the exclusive charge of a competent assistant, who will hold it in proper relation to the other limb, and keep it sufficiently extended. The plaster is then continued until finished. The patient is held in the correct position until the plaster is set. The best bed for him to lie upon is one with a good, firm mattress and strong spring or no spring at all. I believe that the splint should not be put on for several days after the accident. By giving this time the patient recovers largely from the first shock and can better stand to be held erect without so much danger of fainting. I am inclined to suggest, instead of having two strong men to hold the patient erect while the plaster is being put on, that a suitable frame be built to act as two crutches, giving support under the arms. This seems to me would be more steady and the weight of the body could better be held by such a device than by a man on each side of the patient. I have employed the device in one instance with good result.

Before the plaster is set it is advised to cut out a fenestrum over the trochanter of the injured leg so that pressure can be applied in the direction of the neck, thereby holding the fractured ends of the bones more firmly together. The pressure can then be relieved from time to time, and the skin treated to prevent pressure sores. The plaster casing should remain on for three months. During this time the patient can be turned in bed without injury to the limb, and with much advantage to general health. It is in the long confinement in one position that makes the treatment so dangerous to very old or feeble people. Some intercurrent trouble like pneumonia frequently causes failure in the treatment, or death of the patient. It is chiefly this feature that makes fracture of the head of the femur so dreaded in the aged.

